# Safety of Sirolimus in Patients with Tuberous Sclerosis Complex under Two Years of Age—A Bicenter Retrospective Study

**DOI:** 10.3390/jcm12010365

**Published:** 2023-01-03

**Authors:** Dominika Śmiałek, Sergiusz Jóźwiak, Katarzyna Kotulska

**Affiliations:** 1 Department of Pediatric Neurology, Medical University of Warsaw, 02-091 Warsaw, Poland; 2 Research Department, The Children’s Memorial Health Institute, 04-736 Warsaw, Poland; 3 Department of Neurology and Epileptology, The Children’s Memorial Health Institute, 04-736 Warsaw, Poland

**Keywords:** tuberous sclerosis complex, infant, mTOR inhibitor, sirolimus, adverse effect, safety

## Abstract

Background: mTOR inhibitors are a novel pharmacotherapy recommended for subependymal giant astrocytomas, refractory epilepsy, and the treatment of the other clinical manifestations of tuberous sclerosis complex (TSC). Clinical trials on everolimus proved it to be effective and safe in children. Despite its common use in clinical practice, the research on sirolimus is limited. This study is the first to determine and assess the severity of the adverse effects (AEs) of sirolimus administered to children with TSC under two years of age. Methods: We performed a bicenter retrospective data analysis of medical records of individuals with TSC who initiated therapy with sirolimus under the age of two. Results: Twenty-one patients were included in the study. At least one AE was reported in all participants. The most prevalent AEs were anemia, thrombocytosis, and hyperlipidemia. Infections and mouth ulcerations, often reported in the studies on older patients, were infrequent and of mild or moderate grade. Conclusions: Adverse effects associated with sirolimus use in infants and young children with TSC are frequent yet not life- or health-threatening. Further multicenter prospective clinical trials should determine the long-term safety of sirolimus.

## 1. Introduction

Tuberous sclerosis complex (TSC) is a genetic disorder caused by a heterozygous mutation in either of the genes *TSC1* at 9q34 (encoding hamartin) or *TSC2* at 16p13.3 (encoding tuberin), affecting one in 6000 live births [1]. Hamartin and tuberin inhibit the mechanistic target of rapamycin (mTOR), a kinase that regulates protein and lipid synthesis. The mutation in the *TSC1* and *TSC2* genes leads to the overactivation of the mTOR. Its selective activation results in a kinase signaling cascade that increases cell proliferation and growth; hence, multiple benign tumors are formed in different organs [2,3]. Among the most common clinical manifestations of TSC, patients report epilepsy, cortical tubers, subependymal nodules, and subependymal giant cell astrocytomas (SEGA) in the central nervous system, cardiac rhabdomyomas (CRs), as well as renal involvement, such as angiomyolipomas (AML) and renal cysts [4]. Despite their benign character, TSC-associated tumors often become symptomatic and require adequate management. SEGA leads to obstructive hydrocephalus when the tumor grows in the proximity of the interventricular foramen. CRs may block the ventricular flow or cause arrhythmias.

Epilepsy affects approximately 80% of patients with TSC, and in up to 30% of them, seizures become refractory to treatment [2,5]. The earlier appearance of the seizures correlates with worse neurological outcomes and refractoriness, despite adequate antiseizure treatment, even before the onset of seizures [6,7].

Novel pharmacotherapy targeted at the metabolic pathway to inhibit the mTOR kinase has recently been approved in many countries, including EU members, US, and Japan [8]. The mTOR inhibitors changed our view on the management of TSC-associated tumors. Everolimus is approved for treating SEGA, lymphangioleiomyomatosis (LAM), and partial-onset seizures [9,10,11]. As an off-label treatment, it has successfully been used to reduce CRs in children [12,13]. Sirolimus is approved only as LAM therapy, and its efficacy in reducing the frequency and severity of epileptic seizures or treating SEGA or CRs is still being researched. Preclinical studies suggest the mTOR inhibitor’s therapeutic role in autism spectrum disorder, a common psychiatric disorder associated with TSC [14,15].

Randomized clinical trials on everolimus have shown its safety profile in patients with TSC [10,16,17,18]. Despite the lack of research with a similar level of evidence, sirolimus is more often used in clinical practice due to its accessibility. Reports on sirolimus-related adverse effects (AEs), especially in infants and children under two years of age, are limited. These individuals are potential beneficiaries of early antiseizure treatment and SEGA or CRs reduction. Therefore, it raises the question about the safety of sirolimus use in this group of patients.

This study aims to address the gaps in mTOR inhibitor therapy research. We assess the safety of sirolimus in young patients with TSC under the age of two years.

## 2. Materials and Methods

### 2.1. Study Design and Data Collection

The present study is a bicenter retrospective data review of children with TSC treated with mTOR inhibitors between 2014 and 2022.

The data was collected from two hospitals in Warsaw, Poland: the Department of Neurology and Epileptology, The Children’s Memorial Health Institute, and the Department of Child Neurology, the Medical University of Warsaw. We searched the database with the alphanumeric coding of TSC in the ICD-10 list: Q85.1. The data was extracted from patients’ medical records in paper or electronic health records and put into a standardized spreadsheet. The data collection process ended on 30 June 2022.

The inclusion criteria were:

1. Clinical or genetic diagnosis of TSC;
2. Treatment with oral sirolimus before the age of two years;
3. A follow-up at least three months after the initiation of treatment.

The patients were excluded if mTOR inhibitors were introduced after two years of age, the patient was not followed for at least three months, or the applied mTOR inhibitor was everolimus.

The recovered baseline data included:

1. Genetic analysis results: *TSC1* or *TSC2* gene mutation;
2. Sex;
3. Age at the initiation of mTOR inhibitor treatment;
4. Age at the onset of epileptic seizures;
5. Reason for mTOR inhibitor treatment;
6. Whether antiepileptic drugs (ASMs) were included;
7. If the ASM treatment was preventive.

We stored the results of laboratory blood tests, the incidence of infections or mouth ulcerations, sirolimus dose, sirolimus blood concentrations, the patient’s body mass, and the ASMs used. For each patient, the data was analyzed twice, depending on the following:

1. Age: from birth to 6 months of age, 6–12 months, 12–24 months, and 24–36 months of age.
2. Treatment duration (months).

Anemia was defined as either hemoglobin levels below the lower limit of normal (LLN) or red blood cell (RBC) count below the LLN. Hyperlipidemia was determined in case of the elevated blood level of at least one of the below: cholesterol, low-density lipoprotein cholesterol (LDL-C), or triglycerides. Thrombocytosis was considered “mild” for a platelet count (PLT) between 450,000 and 700,00/µL and “moderate” up to 900,000/µL [19]. All the laboratory’s norms for blood tests were adjusted for age.

### 2.2. Study Outcomes

The primary outcome of this study was to assess the safety profile of sirolimus in young children with TSC under the age of two. The data collected at the onset of treatment and then three, six, twelve, and twenty-four months after the initiation focused on the known AEs caused by mTOR inhibitors. Additionally, repetitive alterations in the laboratory results and general outcomes were recorded.

The AEs were graded based on their severity according to Common Terminology Criteria for Adverse Events (CTCAE) v5.0 [20]:

1. Grade 1: Mild
2. Grade 2: Moderate
3. Grade 3: Severe, of medical significance
4. Grade 4: Life-threatening consequences
5. Grade 5: Death related to AE

The interpretation of the laboratory test results was based on the norms provided by the laboratories and adjusted for age.

### 2.3. Statistical Analysis

Continuous variables were calculated; central tendency was reported as mean and median while the variation as standard deviation (SD) and minimum and maximum values, respectively. Quantitative variables were used for sirolimus serum levels calculation. Each AE was assigned a qualitative value (present or not present), and the results were reported as the count and frequency. Analyses were conducted using Statistica 13.3. for Windows.

## 3. Results

Twenty-five patients at both clinical centers started mTOR inhibitor therapy under two years of age. Twenty-one were treated with sirolimus and four with everolimus. One patient began the treatment with sirolimus. After three months, the pharmacotherapy was changed to everolimus due to their inclusion in the national program of everolimus treatment for patients with SEGA not qualified for surgical treatment.

We included all 21 patients treated with sirolimus in this study. Seventeen out of twenty-one patients were treated in the Department of Neurology and Epileptology, The Children’s Memorial Health Institute in Warsaw (17/21, 80.9%). All individuals began pharmacotherapy in the years 2014–2022.

Eight patients were male (8/21, 38.10%), and thirteen were female (13/21, 61.90%). The median age at the onset of sirolimus therapy was 104 days (range 3–656 days). Genetic analysis was performed on 15 patients (15/21, 71.4%). In all 15 patients, a mutation in the *TSC2* gene was found. In addition, three of them carried an additional mutation in the *PKD1* gene (Table 1).

### 3.1. Reasons for mTOR Inhibitor Treatment

The treatment with mTOR inhibitors was initiated due to SEGA in twelve patients (12/21, 57.1%), drug-resistant epilepsy in seven (7/21, 33.3%), rhabdomyomas causing arrhythmia or obstruction of blood flow in five (5/21, 28.6%), and renal angiomyolipomas and retinal hamartomas in one each (1/21, 4.8%). Six patients presented with more than one reason for mTOR inhibitor introduction (6/21, 28.6%) (Table 1).

### 3.2. Sirolimus Dosing

In all patients, sirolimus was administered once a day, orally, in the form of a solution of concentration 1 mg/mL. The protocol for mTOR inhibitor initial dose was 0.5 mg/m^2^/day and varied between 0.01–0.07 mg/kg/day. The dosing regimens were then adjusted according to the sirolimus plasma levels. The targeted levels of sirolimus ranged between 3–4 ng/mL. The median plasma levels of sirolimus throughout the study were 4.34 ng/mL (0.79–13.89 ng/mL).

### 3.3. Data Collection

Data was extracted from medical records collected during the routine check-ups performed at the treatment initiation and three, six, twelve, and twenty-four months after pharmacotherapy initiation.

The clinical tests included laboratory testing, such as complete blood count, lipid profile, liver transaminases, ALT, AST, and creatinine. In some patients, fibrinogen, D- dimers, bilirubin, and gamma-glutamyl transferase (GGTP) were also analyzed. In addition, physical examination, echocardiography, and neuroimaging were performed.

### 3.4. Long-Term mTOR Inhibitor Continuation

Eighteen patients (18/21, 85.71%) continued pharmacotherapy until the end of the follow-up. Nine of them were observed for two years, and nine continued the treatment until the end of data collection, yet the observation period was shorter than two years. Three individuals discontinued the treatment before the end of the follow-up (3/21, 14.29%). The median follow-up period was 12 months.

The reasons for withdrawal were an unsatisfactory response to the treatment; four patients were qualified for neurosurgery, and one began the treatment with cannabidiol. None of the individuals discontinued the treatment due to the AE.

### 3.5. Additional Therapies

During the follow-up, nineteen patients (19/21, 90.5%) received antiseizure treatment. The median number of ASM was two. All of the patients who received ASM were treated with vigabatrin (19/19, 100%), ten with valproic acid (10/19, 52.6%), five with carbamazepine (5/19, 26.3%), five with levetiracetam (5/19, 26.3%), two with clobazam (2/19, 10.5%), and two with topiramate (2/19, 10.5%). Two children received adrenocorticotropic hormone (ACTH), and two patients were on the ketogenic diet.

### 3.6. The Safety Profile of Sirolimus Therapy

At least one adverse effect was reported in all patients (21/21, 100.0%) (Table 2). The number of tested patients varied for each AE, which is specified in the corresponding column in Table 2.

Twelve individuals began the treatment between birth and six months of age. In this group, the most common AE was anemia in eleven (11/12, 91.67%), hyperlipidemia in ten (10/12, 83.33%), with the most frequent being hypertriglyceridemia in seven (7/12, 58.33%), and elevated PLT in seven (7/12, 58.33%). One patient had low PLT (1/12, 8.33%).

Fourteen patients continued or started treatment with sirolimus between six and twelve months of age. The most common side effects were anemia in eight (8/14. 57.14%), hyperlipidemia in eleven (11/13, 84.62%), and elevated PLT in five (5/14, 35.71%).

Fourteen patients were on treatment between one and two years of age. Nine of them had anemia (9/14, 64.29%), thirteen had hyperlipidemia (13/13, 100.0%), and eleven had elevated PLT (11/14, 78.57%). Two patients with hyperlipidemia were on the ketogenic diet at that time.

The data was available for ten patients two years and older. The most common AE was elevated PLT in seven (7/10, 70.0%), anemia in five (5/10, 50.0%), and hyperlipidemia in five (5/8, 62.5%).

D-dimers and fibrinogen were rarely tested despite high PLT.

Throughout the follow-up, infections were reported in 16.67% up to 50% of the patients, most common in individuals two years and older. On the other hand, neutropenia was reported in 10% up to 41.67% of the patients, and it was most prevalent in patients under six months of age. One individual required hospitalization several times. One patient’s parents noticed a pattern between higher serum sirolimus levels and the frequency of infections.

Three cases of mouth ulcerations were reported, two in patients younger than six months (2/11, 18.18%) and one in a patient older than two years (1/10, 10.0%).

The majority of adverse effects were grade 1 and 2 according to the CTCAE. All patients with anemia reported hemoglobin levels between 8.5 g/dL and the LLN for the respective age group.

Elevated PLT in most participants was between 450 and 600/µL. One child reported a PLT count of 745/µL.

None of the participants in the study had to be hospitalized due to hyperlipidemia. In most patients with elevated LDL-C, the levels were between 130 mg/dL and 280 mg/dL. Hypertriglyceridemia was reported to be between 150 mg/dL and 300 mg/dL. One patient had a triglyceride level of 750 mg/dL. The individual was on the ketogenic diet at that time; a modification of proportions led to the normalization of lipid levels. The levels exceeded 1000 mg/dL in none of the participants.

One patient was hospitalized two times due to infections: varicella and not identified bacterial infection treated with antibiotics. Both times the patient presented with fever and epileptic seizures. No AEs grade 4 or 5 were reported.

**Figure 1:**
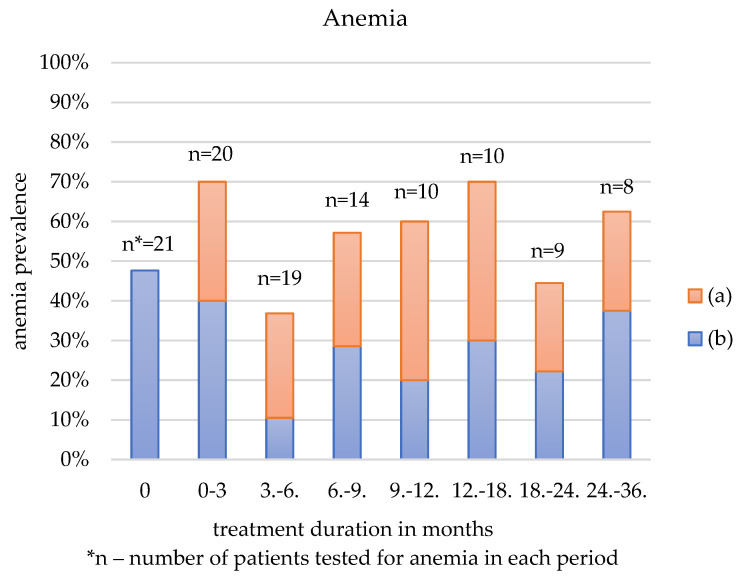
Anemia according to the treatment duration in patients with: (a) no history of anemia prior to sirolimus treatment, (b) a history of anemia prior to sirolimus treatment.

**Figure 2:**
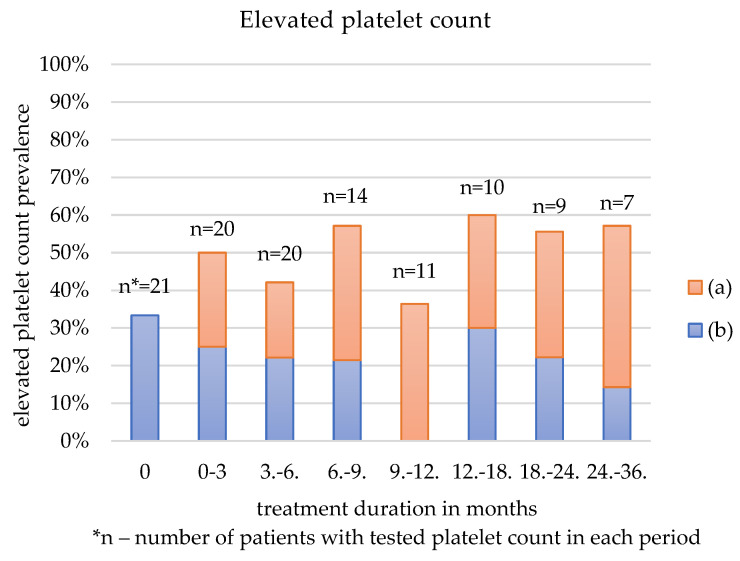
Elevated platelet count depending on the treatment duration in patients with: (a) platelet count within normal ranges prior to sirolimus treatment, (b) thrombocytosis prior to sirolimus treatment.

**Figure 3:**
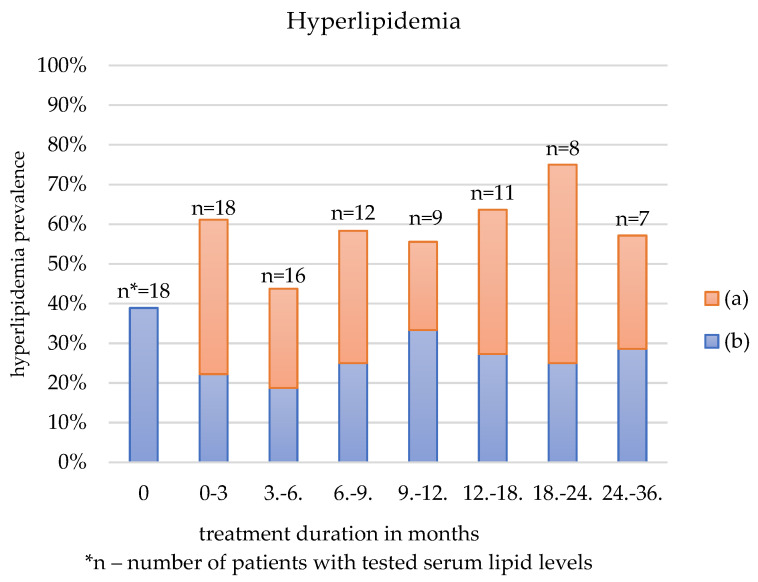
Hyperlipidemia depending on the treatment duration in patients with: (a) normal lipid blood levels prior to sirolimus treatment, (b) hyperlipidemia prior to sirolimus treatment.

Table 3 and Figure 1, Figure 2 and Figure 3 show the three most common AEs: anemia, thrombocytosis, and hyperlipidemia, depending on the duration of sirolimus administration. The figures depict all patients who reported any of those AEs: blue-coded individuals with a history of laboratory alternation prior to sirolimus treatment and orange-coded patients with no record of such disorder.

The prevalence of anemia, thrombocytosis, and hyperlipidemia was first calculated for all patients who had the factor tested. Then the frequency was estimated among the patients with normal hemoglobin and RBC, PLT, or serum lipids prior to the sirolimus administration.

Anemia was reported in 40–70% of individuals during the follow-up. This AE was observed for the first time in the first three months of pharmacotherapy in more than half of the patients.

Thrombocytosis was reported for the first time in almost 40% of the patients in the first three months after starting the treatment. After six months of pharmacotherapy, in this group, the frequency of elevated PLT rose to 70%; it remained elevated in over two-thirds of the patients until the end of the follow-up.

In the first three months of treatment, lipid blood levels were elevated in more than 60% of the patients. This number dropped to 40% in the following three months. Regardless of the group size, hyperlipidemia remained present in 40–60% of individuals during the follow-up.

## 4. Discussion

Randomized clinical trials, EXIST-1, and EXIST-3 proved everolimus as effective and safe in young children [16,21,22]. Other studies and reports are consistent with those findings [9,10,11]. Although sirolimus is used more often than everolimus, its efficacy and safety have only been assessed in studies with a smaller level of evidence in older children and adults with TSC [23,24,25,26,27]. This is the first study to report AEs caused by sirolimus use in the youngest patients under the age of two.

The AEs were observed in all participants during the follow-up, yet they tended not to be severe and did not lead to pharmacotherapy discontinuation. Clinical trials on sirolimus used in older patients with TSC also found it to cause low-grade AEs, grade 1 or 2 [10,16,28]. Children under three years of age treated with everolimus during the EXIST study reported AEs of severity grade 1 or 2 as well. Only one patient from this group withdrew from the treatment due to an AE [16].

In research studies on sirolimus, the target serum levels range between 4–20 ng/mL, typically 5–15 ng/mL [9,26,28,29]. In this study, blood trough levels remained in the lower spectrum of those ranges, which may contribute to the lower severity of the AEs.

All the included participants started the treatment with mTOR inhibitors due to SEGA, CRs, or refractory seizures, which are the most common causes according to Krueger et al. and Saffari et al. [10,17,28]. In every individual with genetic testing performed, a *TSC2* mutation was found, which is known to correlate with more severe TSC symptoms [30]. As a result of the severity of the disease, six individuals were treated with mTOR inhibitors due to more than one cause.

Hyperlipidemia, anemia, and thrombocytosis were the most frequently reported AEs during the follow-up, regardless of age or the duration of the treatment.

### 4.1. Hyperlipidemia

The majority of participants reported mild or moderate hyperlipidemia, especially hypertriglyceridemia, during the follow-up. It is consistent with previous clinical trials, which found mTOR inhibitors lead to elevated, yet not life-threatening, serum lipid levels [4,12,13,16]. Hyperlipidemia caused by mTOR inhibitors is repeatable, reversible, and dose-dependent [31]. As we did not follow the patient’s lipid levels after therapy discontinuation, the reversibility of hyperlipidemia was not assessed.

Two patients with the highest blood lipid levels were on a ketogenic diet at that time, also known to cause hyperlipidemia [32]. In those children, the lipid-to-nonlipid ratio was modified, as according to Fang et al., diet modification should improve the laboratory results [33]. After the adjustment, the lipid levels lowered; however, they remained elevated compared to the normal ranges.

### 4.2. Anemia

Anemia is one of the most often reported sirolimus-induced AEs [10,25,26]. In this study, anemia was the most frequent among the youngest individuals between birth and six months of age. Sirolimus affects iron homeostasis, leading to mild microcytic anemia [34]. However, in this specific group of patients, anemia may have been partially due to physiological anemia, which occurs in all infants between ten and twelve weeks of age, being the most severe in preterm infants [35]. Table 3 represents the data not biased by the physiological anemia, as it is based on the duration of treatment, not the age. This data demonstrates that anemia is frequently reported regardless of the age of the participants.

According to the literature, mTOR inhibitor discontinuation due to anemia is rare [25,26]. During the follow-up in this study, no radical interventions were undertaken. As the AE was of grade 1 or 2 in most cases, iron supplementation and dietary modifications were recommended.

### 4.3. Thrombocytosis

Sirolimus use in human trials often leads to thrombocytopenia [24,25,36,37,38]. According to Busca et al., low platelet count correlates significantly with sirolimus through concentrations, yet they tend to be much higher than the concentrations maintained in our research [39]. In this study, only one patient reported thrombocytopenia.

Elevated PLT was frequent in all age groups, yet in none of the individuals did it lead to any further clinical consequences, nor did it require intervention. No literature on the sirolimus impact on PLT elevation was found. Interestingly, due to its immunosuppressive effect, sirolimus can be applied as rescue therapy for thrombocytopenia in primary antiphospholipid syndrome [40].

### 4.4. Infections

Sirolimus belongs to a class of immunosuppressants and has proven its efficacy in solid organ transplantation. Its immunosuppressive effect may increase the susceptibility to infections, in particular pneumonitis or stomatitis [41,42,43,44]. Despite being reported frequently by patients treated with sirolimus, infections tend to be mild and often do not lead to hospitalization [17,23,28]. During the follow-up, one individual had to be hospitalized due to an infection to provide constant surveillance. No emergency interventions were undertaken. According to Krueger et al. and Guemes et al., infections often lead to resignation from the treatment, yet in our study, none of the participants withdrew due to this cause [27,28].

Stomatitis or mouth ulcerations are likely dose-dependent and often reported in older children, requiring short-term or complete treatment withdrawal [10,16,18]. During the follow-up, they were reported sporadically and, being of low severity grade, required only sirolimus discontinuation for a few days.

### 4.5. Discontinuation

Few interventions were needed to minimalize the AEs caused by sirolimus use. In most cases, a dose adjustment or temporary discontinuation had to be undertaken. Sirolimus dosing alternations may cause imprecise serum level calculations and require further modifications to the dosing scheme.

The typical drop-out rate in the studies on mTOR inhibitors in patients with TSC is 0–5% [17,45,46]. In our study, almost 15% of the participants resigned from pharmacotherapy with sirolimus. However, an AE was not the reason for the discontinuation in any of them. Most patients resigned due to unsatisfactory results and treatment alternatives available.

## 5. Limitations of the Study

The study was a retrospective data collection, which may be subject to patient, parent, and physician recall bias and differences in the history-taking methodology. The relatively small number of participants assessed in this analysis can lead to conclusions that should not be applied to all patients.

## 6. Conclusions

The analysis is the first to assess the safety of sirolimus in infants and young children under two years of age. In the EU and the US, mTOR inhibitors are currently recommended as a treatment for distinct TSC clinical manifestations. Clinical trials have proved everolimus’ efficacy and safety, yet the research on sirolimus is limited. The innovative character of this study is emphasized by the size of the cohort, which included all patients from two clinical centers specializing in child neurology in Poland.

Adverse effects associated with sirolimus use in infants and young children with TSC are common yet not life- or health-threatening. The most frequent AEs in this group of patients are anemia, hyperlipidemia, and thrombocytosis. Mouth ulcerations or stomatitis are not common, and infections tend to be mild. Sirolimus appears to be safe and well tolerated in young patients with TSC.

Further prospective studies are recommended to support the findings.

## Figures and Tables

**Table 1:** Patients’ characteristics.

Variable	Value (%)
	N = 21
Sex	
Female	13 (61.9)
Male	8 (38.1)
TSC mutation	
*TSC1*	0 (0)
*TSC2*	15 (71.4)
*PKD1*	3 (14.3)
Not studied	6 (28.6)
Reason for mTORi ^1^ treatment	
Cardiac rhabdomyomas	5 (28.6)
SEGA	12 (57.1)
Renal AML	1 (4.8)
Retinal hamartomas	1 (4.8)
Epilepsy	7 (33.3)
Age at the initiation of mTORi treatment (days)	
Mean (SD)	211.9 (233.2)
Median [Min, Max]	104 [3, 656]
mTORi treatment follow-up duration (months)	
Mean (SD)	16 (8.7)
Median [Min, Max]	22 [3, 24]
Antiepileptic treatment	
Yes	19 (90.5)
No	2 (9.5)
Number of ASM ^2^	
Mean (SD)	2.0 (1.1)
Median [Min, Max]	2.0 [0, 3.0]
Preventive antiepileptic treatment	
Yes	7 (33.3)
No	14 (66.7)

^1^ mTORi—mTOR inhibitor. ^2^ ASM—antiseizure medication.

**Table 2:** Sirolimus adverse effects depending on age.

Adverse Effect|Age	0–6 Months	6–12 Months	12–24 Months	24–36 Months
	N ^1^ (%)	N (%)	N (%)	N (%)
Hyperlipidemia	10/12 (83.33)	11/13 (84.62)	13/13 (100)	5/8 (62.5)
Hypercholesterolemia	8/12 (66.67)	5/12 (41.67)	8/13 (61.54)	3/8 (37.5)
Elevated LDL	5/11 (45.45)	5/12 (41.67)	7/13 (53.85)	2/7 (28.57)
Hypertrigliceridemia	7/12 (58.33)	8/12 (66.67)	10/13 (76.92)	3/7 (42.86)
Anemia	11/12 (91.67)	8/14 (57.14)	9/14 (64.29)	5/10 (50)
Thrombocytosis	7/12 (58.33)	5/14 (35.71)	11/14 (78.57)	7/10 (70)
Neutropenia	5/12 (41.67)	3/14 (21.43)	5/14 (35.71)	1/10 (10)
Elevated D-dimers	2/2 (100)	0/1 (0)	1/2 (50)	0/0 (0)
Elevated bilirubin	3/7 (42.86)	0/4 (0)	0/4 (0)	1/6 (16.67)
Low ALT	4/12 (33.33)	4/13 (30.77)	6/12 (50)	4/7 (57.14)
Elevated AST	2/12 (16.67)	1/13 (7.69)	4/12 (25)	2/7 (28.57)
Elevated fibrinogen	0/6 (0)	1/2 (50)	1/3 (33.33)	0/1 (0)
Infections	2/12 (16.67)	3/13 (23.08)	5/13 (38.46)	5/10 (50)
Mouth ulcers	2/11 (18.18)	0/14 (0)	0/13 (0)	1/10 (10)

^1^ N—number of patients who reported the adverse effect divided by the number of patients who were tested for the adverse effect.

**Table 3:** Anemia, thrombocytosis and hyperlipidemia as an adverse effect depending on the duration of treatment.

	Anemia		Elevated Platelet Count		Hyperlipidemia	
Duration of the Treatment (Months)	All Patients	No History of Anemia Prior to Sirolimus	All Patients	No History of Elevated PLT ^1^ Prior to Sirolimus	All Patients	No History of Hyperlipidemia Prior to Sirolimus
	N ^2^ (%)	N (%)	N (%)	N (%)	N (%)	N (%)
0	10/21 (47.62)	0/11 (0)	7/21 (33.33)	0/14 (0)	7/18 (38.89)	0/11 (0)
0–3	14/20 (70)	6/11 (54.55)	10/20 (50)	5/13 (38.46)	11/18 (61.11)	7/11 (63.64)
3–6	7/19 (36.84)	5/10 (50)	8/20 (42.11)	4/11 (36.36)	7/16 (43.75)	4/10 (40)
6–9	8/14 (57.14)	4/8 (50)	8/14 (57.14)	5/7 (71.43)	7/12 (58.33)	4/7 (57.14)
9–12	6/10 (60)	4/6 (66.67)	4/11 (36.36)	4/6 (66.67)	5/9 (55.56)	2/5 (40)
12–18	7/10 (70)	4/5 (80)	6/10 (60)	3/3 (100)	7/11 (63.64)	4/6 (66.67)
18–24	4/9 (44.44)	2/5 (40)	5/9 (55.56)	3/4 (75)	6/8 (75)	4/6 (66.67)
24–36	5/8 (62.50)	2/4 (50)	4/7 (57.14)	3/3 (100)	4/7 (57.14)	2/4 (50)

^1^ PLT—platelet count. ^2^ N—number of patients who reported the adverse effect divided by the number of patients who were tested for the adverse effect.

## Data Availability

The data generated and/or analysed in this study are available from the corresponding author upon reasonable request.
